# PUST Cafeteria Food Image Dataset: Real-world Bangladeshi meal-platter images with bounding-box and polygon annotations

**DOI:** 10.1016/j.dib.2026.113262

**Published:** 2026-09-13

**Authors:** Marufa Yeasmin, Md. Razu Ahmed, Protiva Rani Kundu, Jannatul Mauya, Md. Shamim Reza

**Affiliations:** a Deep Statistical Learning and Research (DSLR) Lab, Department of Statistics and Data Science, Pabna University of Science and Technology, Pabna, 6600, Bangladesh; b Department of Statistics and Data Science, Pabna University of Science and Technology, Pabna, 6600, Bangladesh

**Keywords:** Bangladeshi food, Cafeteria meals, Food image dataset, Object detection, Instance segmentation, YOLO annotations, COCO annotations

## Abstract

The PUST Cafeteria Food Image Dataset is a real-world image resource developed for object detection and instance segmentation of mixed Bangladeshi cafeteria meals. The source collection comprises 720 meal-platter photographs acquired between 1 August and 5 September 2025 at the Central Cafeteria of Pabna University of Science and Technology, Bangladesh. Images were captured using a Realme GT Master Edition and an OPPO A92 during routine cafeteria operations without standardized illumination, camera position, camera-to-platter distance, background, plate configuration, or food arrangement. Each identifiable food instance was manually annotated in Roboflow using a bounding box and polygon vertices delineating its visible region. Following auto-orientation, resizing, selective augmentation, light class balancing, and quality review, 1,728 images containing 6,297 annotated food instances across 16 classes were retained in the final processed dataset. The dataset was organized into training, validation, and test subsets comprising 1,512, 108, and 108 images, respectively. Annotations are provided in YOLO-compatible TXT and COCO JSON formats, supporting reuse across object-detection and instance-segmentation frameworks. The dataset can be reused for regional food-image analysis, comparative benchmarking, transfer learning, domain adaptation, and intelligent food-service research.

Specifications Table

**Table:** 

Subject	Computer Vision and Artificial Intelligence
Specific subject area	Food image analysis, multi-class object detection, and instance segmentation of real-world Bangladeshi cafeteria meals
Type of data	Meal-platter images in JPG format with corresponding annotation files in YOLO-compatible TXT and COCO JSON formats. The dataset contains bounding-box annotations for object detection and polygon-based instance masks for instance segmentation tasks.
Data collection	Meal-platter images were collected from 1 August to 5 September 2025 at the Central Cafeteria of Pabna University of Science and Technology, Pabna-6600, Bangladesh. The source collection consisted of 720 high-resolution original images captured using the rear cameras of two smartphones: a Realme GT Master Edition and an OPPO A92. Images were acquired under natural cafeteria conditions without controlled illumination, fixed camera position, standardized camera-to-platter distance, background, plate configuration, or food arrangement, thereby representing realistic meal-serving environments. The annotations were manually prepared by two annotators using the Roboflow platform, with each food instance assigned appropriate class labels and annotated using bounding boxes and polygons for object detection and instance segmentation. The images were auto-oriented and resized to 640 × 640 pixels. To prevent data leakage, dataset partitioning was performed at the original image level before augmentation. The 720 original images were first divided into three independent subsets: training (504 images), validation (108 images), and test (108 images). Data augmentation was then applied exclusively to the training subset, generating additional training samples while keeping the validation and test subsets unchanged. Therefore, no augmented derivative of any training image was included in the validation or test partitions. Following selective augmentation, light class balancing, quality review, and dataset refinement, the final processed dataset contained 1728 images with 6297 annotated food instances across 16 classes. The final dataset consisted of 1512 training images with 5493 annotated food instances, 108 validation images with 395 annotated food instances, and 108 test images with 409 annotated food instances. The released annotations were prepared in YOLO-compatible TXT and COCO JSON formats to support both object detection and instance segmentation workflows.
Data source location	Institution: Pabna University of Science and Technology Specific data-collection site: Central Cafeteria City/Town/Region: Pabna Postal code: 6600 Country: Bangladesh
Data accessibility	Repository name: Mendeley Data Dataset title: PUST Cafeteria Food Image Dataset: Real-World Bangladeshi Meal-Platter Images with Bounding-Box and Polygon Annotations Data identification number: 10.17632/fn6yhzjz83.2 Direct URL to data: https://data.mendeley.com/datasets/fn6yhzjz83/2 Instructions for accessing these data: Open the dataset record, select “Download All Files,” and extract the downloaded files to access the original and processed meal-platter images and corresponding annotations for YOLO object detection, YOLO instance segmentation, and COCO-compatible workflows.
Related research article	None

## 1 Value of the Data

• The dataset provides a real-world collection of 720 original cafeteria meal-platter photographs and a final processed release of 1728 images containing 6297 annotated food instances across 16 commonly served Bangladeshi food classes. It expands the availability of region-specific, spatially annotated food datasets for computer-vision research, particularly for mixed South Asian meal scenarios.
• Each visually distinguishable food instance is annotated using both bounding boxes and polygon-based segmentation masks, enabling applications in object detection, instance segmentation, food-instance localization, multi-task learning, and comparative evaluation of vision models under a unified class taxonomy.
• The images were captured under natural cafeteria operating conditions, incorporating realistic variations in illumination, viewpoint, background, plate arrangement, food presentation, overlap, and partial occlusion. These characteristics make the dataset suitable for evaluating model robustness in complex multi-food environments rather than controlled laboratory settings.
• The dataset provides standardized YOLO-compatible TXT and COCO JSON annotation formats with predefined training, validation, and test partitions. This reduces preprocessing requirements and supports reproducible benchmarking across different object-detection and instance-segmentation frameworks, including lightweight and deployment-oriented architectures.
• The dataset can support research in computer vision, food computing, artificial intelligence, and intelligent food-service systems, including regional food recognition, food-instance counting, transfer learning, domain adaptation, cross-dataset evaluation, and class-wise performance analysis. When combined with external validated information sources, such as cafeteria pricing databases or nutritional reference datasets, it may further support future development of image-assisted food-service and dietary-analysis applications.

## 2 Background

Food-image datasets support the development and evaluation of computer-vision methods for food classification, localization, and segmentation. Food-101 established a widely used benchmark comprising 101,000 images across 101 food categories; however, it was developed primarily for image-level classification and does not provide object-level spatial annotations for identifying individual foods within multi-item meal scenes [1]. Culturally representative datasets are also important because food composition, preparation, appearance, and presentation vary across regional cuisines. Several datasets have improved the representation of Bangladeshi foods in computer-vision research. The Traditional Bengali Food Image dataset contains 2835 images across seven traditional food categories and was developed for image-level classification [2]. A subsequent Bangladeshi dessert dataset provides 4118 original images representing 27 dessert categories, but it similarly focuses on classification within a restricted food group [3]. More recently, FoodBD introduced 3523 smartphone-captured Bangladeshi meal images across 67 food categories with polygon-based food-region annotations; 1837 images additionally include expert-estimated nutritional information [4]. These resources address important aspects of regional food recognition, although their acquisition settings, annotation structures, and intended applications differ from those of a focused university-cafeteria dataset providing corresponding annotations for object detection and instance segmentation. Spatial annotations are particularly important for mixed-meal images because adjacent food items may have irregular boundaries, similar colours and textures, direct physical contact, overlap, and partial occlusion. UNIMIB2016 comprises 1027 canteen-tray images containing 3616 food instances across 73 classes, with individual food regions manually delineated using polygonal boundaries [5]. VIPER-FoodNet provides approximately 15,000 images across 82 food categories with food-object bounding boxes and class labels [6], whereas UEC-FoodPix Complete contains 10,000 food images with segmentation masks, including 9000 masks manually refined from the preceding dataset version [7]. These resources demonstrate the value of spatially annotated data for analyzing complex food scenes, but their cuisine coverage and acquisition environments do not specifically represent mixed meals served in Bangladeshi university cafeterias.

The PUST Cafeteria Food Image Dataset [8] was developed as a complementary, setting-specific resource for this context. It was derived from 720 original meal-platter photographs acquired under natural conditions at the Central Cafeteria of Pabna University of Science and Technology, Bangladesh. Following image preprocessing, selective augmentation, light class balancing, quality review, and dataset refinement, the final processed dataset contains 1728 images with 6297 annotated food instances across 16 commonly served cafeteria food classes. Each distinguishable food instance is represented by both a bounding-box annotation for object detection and a polygon-based instance annotation for segmentation, provided in YOLO-compatible formats. This paired annotation structure enables the development and comparative evaluation of object-detection and instance-segmentation models using a common image collection, annotation framework, and unified class taxonomy. The dataset complements existing food-image resources by providing spatially annotated, real-world Bangladeshi cafeteria meal scenes captured under natural acquisition conditions.

## 3 Data Description

### 3.1 Dataset composition and contents

The PUST Cafeteria Food Image Dataset [8] is a real-world image resource developed for object detection and instance segmentation of mixed Bangladeshi cafeteria meals. The source photographs were collected under natural operating conditions at the Central Cafeteria of Pabna University of Science and Technology, Bangladesh, and include both single-item and multi-item meal platters representing realistic cafeteria serving scenarios. The source collection consisted of 720 original meal-platter photographs. Each image was manually annotated in Roboflow, where every visually distinguishable food item was assigned a class label and represented using both bounding-box annotations for object detection and polygon-based annotations for instance segmentation. The processed images were auto-oriented and standardized to 640 × 640 pixels. To ensure reliable evaluation and prevent data leakage, the dataset was partitioned at the original image level before augmentation. The original collection was divided into three independent subsets: 504 training images, 108 validation images, and 108 test images. Data augmentation and selective light class balancing were subsequently applied only to the training subset. Therefore, augmented derivatives of training images were restricted exclusively to the training partition, while validation and test images remained unchanged. Following preprocessing, selective augmentation, light class balancing, quality review, and dataset refinement, the final processed dataset comprises 1728 images with 6297 annotated food instances across 16 cafeteria food classes. The final dataset was organized into 1512 training images, 108 validation images, and 108 test images. The processed images are provided in JPG format, while corresponding annotations are available in YOLO-compatible TXT and COCO JSON formats. Each food instance in the dataset is associated with a class identifier, bounding-box coordinates, and polygon vertex coordinates describing the visible food region. Images containing multiple food items include separate annotation records for each distinguishable instance. Table 1 summarizes the dataset composition and technical characteristics, while representative single-item and multi-item meal-platter examples are presented in Fig. 1. The food-class distribution, annotation formats, directory organization, and dataset preparation procedures are described in the following Section 4.

**Table 1:** Composition and technical characteristics of the PUST Cafeteria Food Image Dataset.

Dataset characteristic	Description or value
Acquisition setting	Central Cafeteria, Pabna University of Science and Technology, Bangladesh
Source collection	720 original meal-platter photographs
Final processed dataset	1728 images
Annotated food instances	6297
Food classes	16
Original training images before augmentation	504 images
Validation subset	108 images
Test subset	108 images
Final training subset after augmentation	1512 images
Annotated instances in the train subset	5493
Annotated instances in the validation subset	395
Annotated instances in the test subset	409
Processed image dimensions	640 × 640 pixels
Image file format	JPG
Detection annotations	Class labels and bounding-box coordinates
Segmentation annotations	Class labels and polygon coordinates
Annotation file formats	YOLO-compatible TXT and COCO JSON
Metadata files	Class taxonomy, dataset statistics, and image-source lineage mapping

**Fig. 1:**
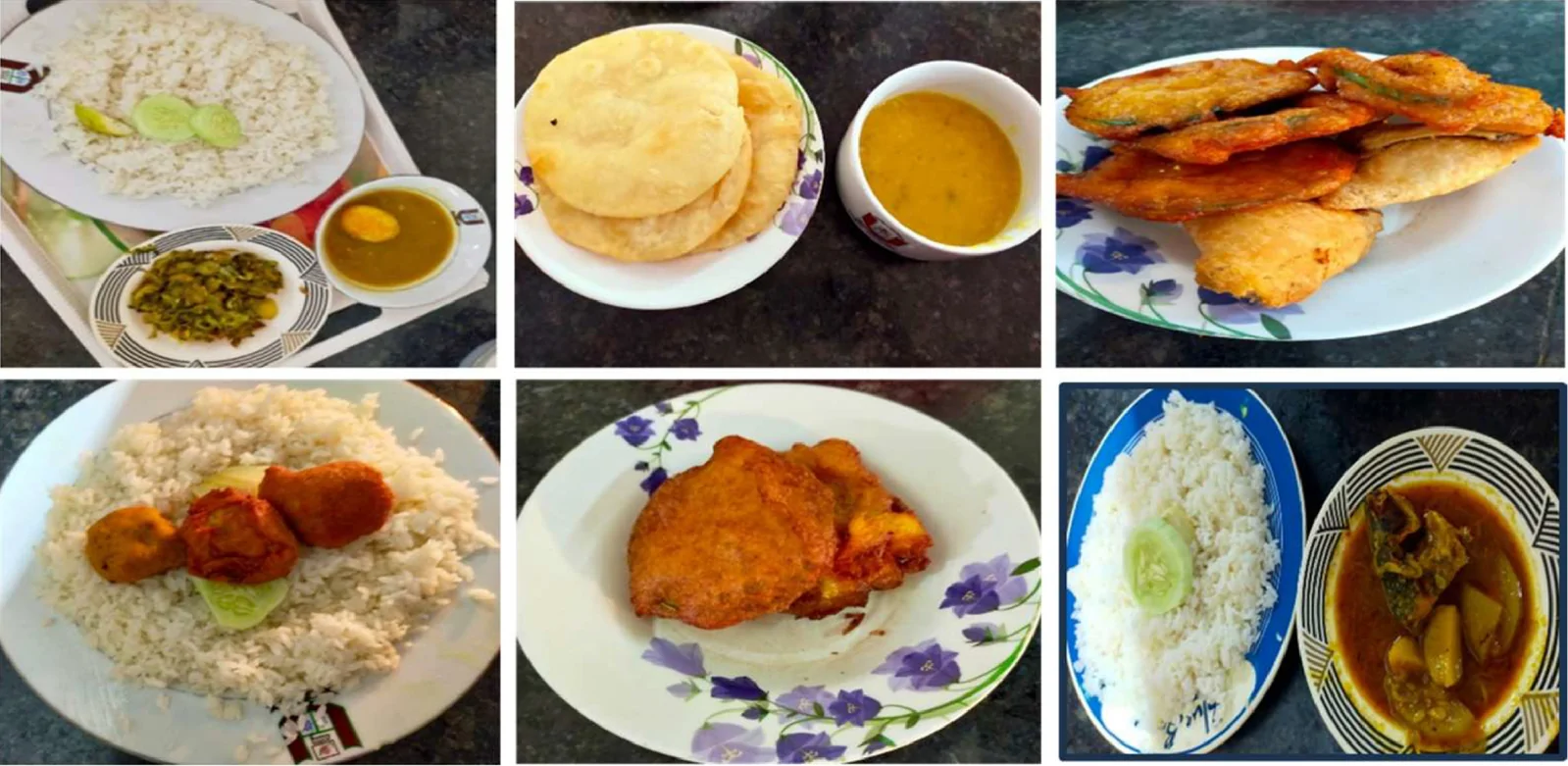
Representative single-item and multi-item meal-platter images from the PUST Cafeteria Food Image Dataset.

### 3.2 Food-class taxonomy and instance distribution

The dataset contains 16 food classes representing staple foods, curries, side dishes, snacks, beverages, and other prepared items commonly served at the PUST Central Cafeteria. The class taxonomy was developed to represent visually distinguishable food items present in real-world cafeteria meals. Several class labels are transliterated Bengali food names; therefore, brief English descriptions are provided to improve accessibility for international users. These descriptions serve as explanatory metadata and do not replace the original class labels used in the annotation files. Before applying training-only augmentation and selective light class balancing, the initial annotated instance distribution was analyzed to characterize the natural variation among the 16 food categories. Table 2 presents the class identifiers, dataset labels, English descriptions, and the distribution of annotated food instances across the original training, validation, and test subsets. Fig. 2 represents the class-wise distribution of annotated food instances across the 16 food classes before training-only augmentation and light class balancing. The original dataset contained 2635 annotated food instances, including 1828 training instances, 395 validation instances, and 409 test instances. The initial distribution showed considerable variation among food categories. Salad (648 instances) and rice (616 instances) were the most frequently represented classes, whereas singara (23 instances), matha (26 instances), and vegetable_roll (28 instances) had relatively fewer instances. This natural imbalance motivated the application of selective augmentation-based light class balancing during training data preparation, while preserving the original validation and test subsets.

**Table 2:** Class-wise distribution of annotated food instances before training-only augmentation and light class balancing.

Class ID	Dataset label	English equivalent / description	Train instances	Validation Instances	Test instances	Total
0	chaa	Tea	24	6	6	36
1	chola	Chickpea curry	75	16	13	104
2	chop	Croquette	268	70	70	408
3	daal	Lentil curry	53	12	14	79
4	egg	Egg	71	9	10	90
5	fish	Fish	26	8	6	40
6	mashed_potato	Mashed potato	120	18	21	159
7	matha	Buttermilk	18	2	4	24
8	meat	Meat curry	46	7	9	62
9	puri	Fried puff bread	110	36	39	185
10	rice	Rice	435	90	91	616
11	ruti	Flatbread	25	6	1	32
12	salad	Salad	451	98	99	648
13	singara	Singara	16	0	7	23
14	vegetable	Vegetable	69	12	17	98
15	vegetable_roll	Vegetable roll	21	5	2	28
Total			1828	395	409	2635

Note. Original Bengali transliterated labels were retained to preserve annotation compatibility, while English equivalents were provided for international accessibility.

**Fig. 2:**
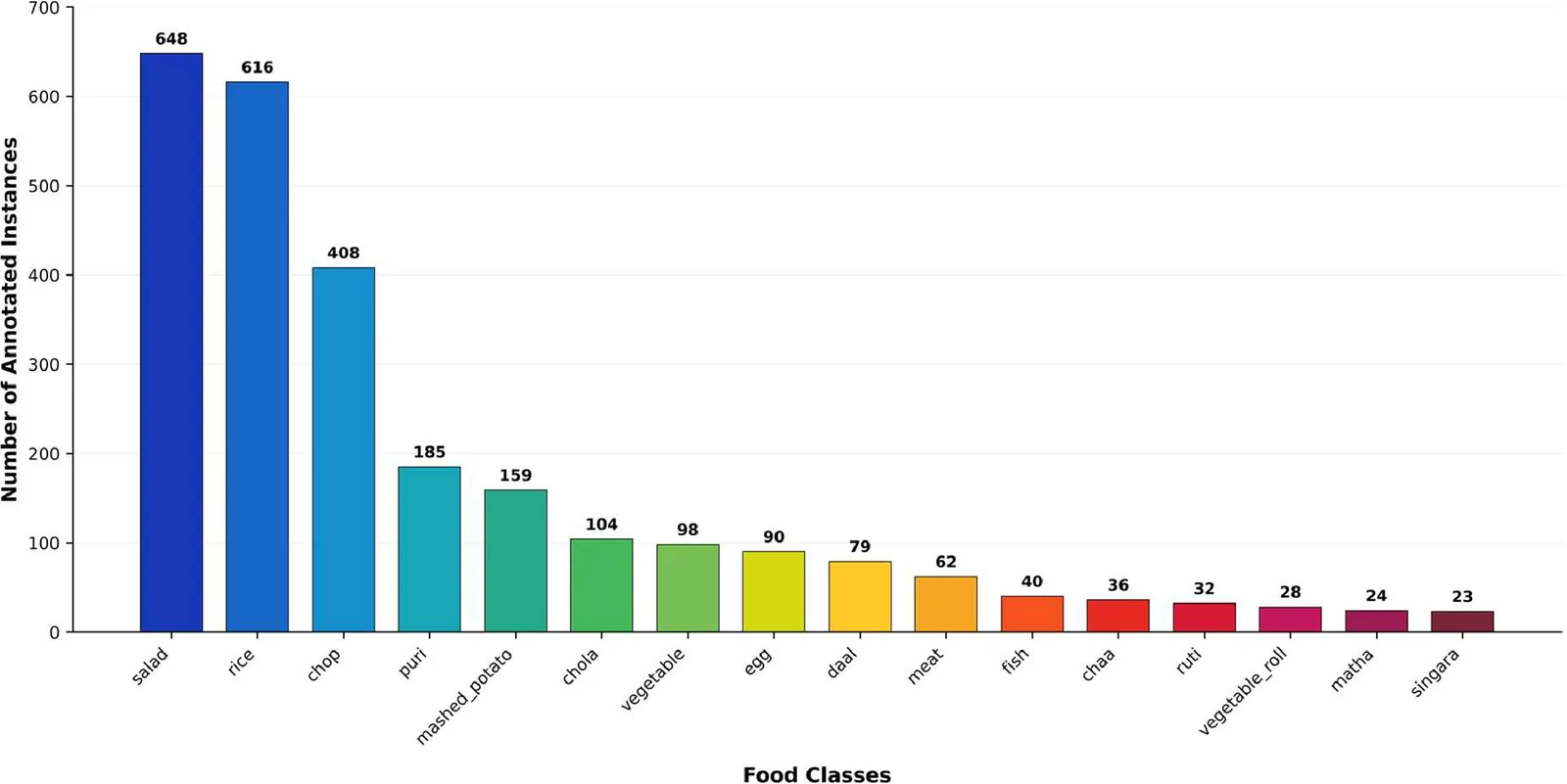
Class-wise distribution of annotated food instances across the 16 food classes before training-only augmentation and light class balancing.

Training-only augmentation and selective light class balancing were subsequently applied to improve the representation of relatively underrepresented classes while maintaining the original validation and test subsets. The resulting class-wise distribution of the released dataset after training-only augmentation and light class balancing is presented in Table 3 and Fig. 3.

**Table 3:** Class-wise distribution of annotated food instances after training-only augmentation and light class balancing.

Class ID	Dataset label	English equivalent / description	Train instances	Validation Instances	Test instances	Total
0	Chaa	Tea	75	6	6	87
1	Chola	Chickpea curry	225	16	13	254
2	Chop	Croquette	804	70	70	944
3	Daal	Lentil curry	159	12	14	185
4	Egg	Egg	213	9	10	232
5	Fish	Fish	78	8	6	92
6	mashed_potato	Mashed potato	360	18	21	399
7	Matha	Buttermilk	60	2	4	66
8	Meat	Meat curry	138	7	9	154
9	Puri	Fried puff bread	330	36	39	405
10	Rice	Rice	1305	90	91	1486
11	Ruti	Flatbread	75	6	1	82
12	Salad	Salad	1353	98	99	1550
13	Singara	Singara	48	0	7	55
14	Vegetable	Vegetable	207	12	17	236
15	vegetable_roll	Vegetable roll	63	5	2	70
Total			5493	395	409	6297

**Fig. 3:**
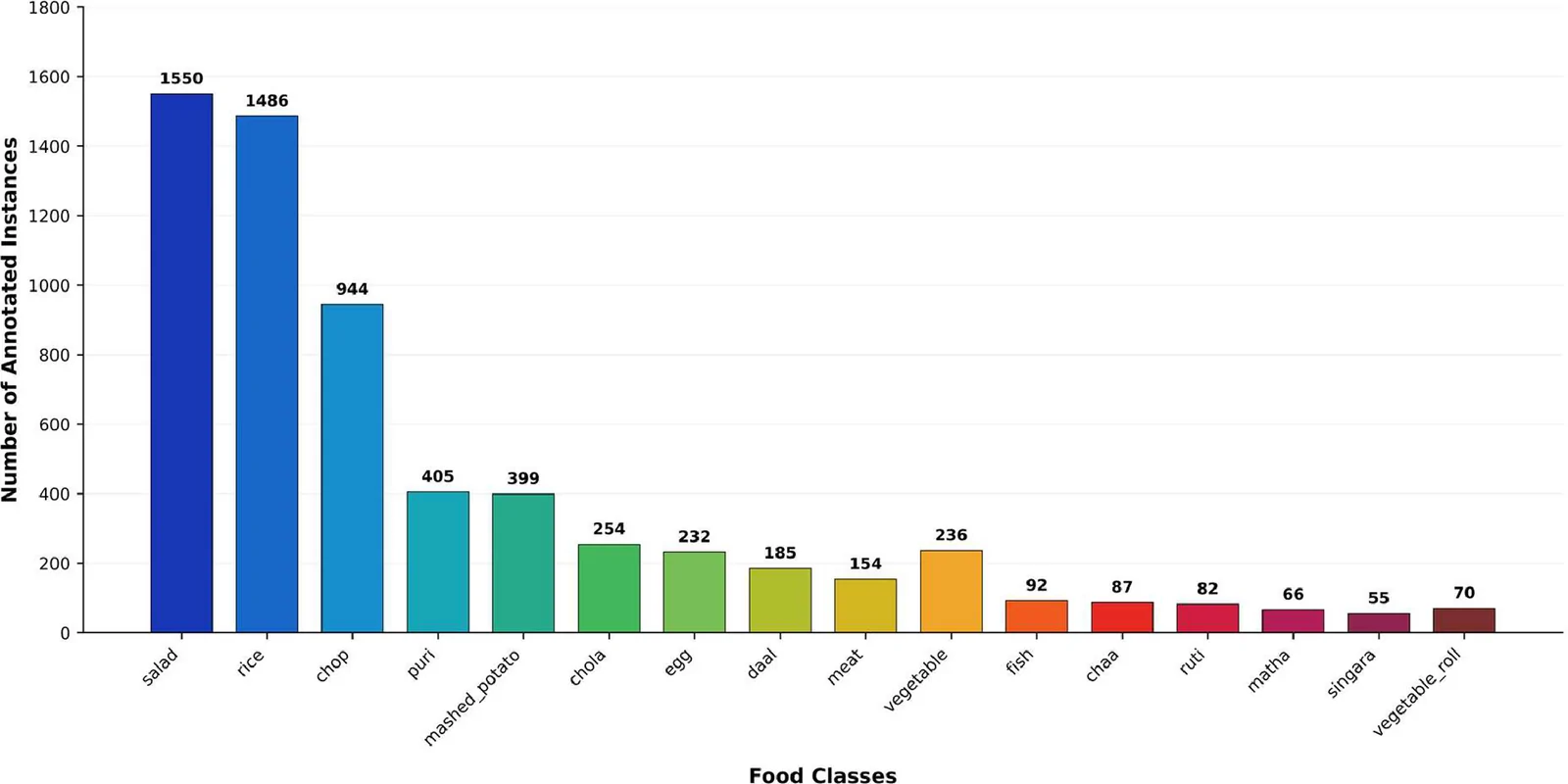
Class-wise distribution of annotated food instances after training-only augmentation and light class balancing.

After augmentation and refinement, the final dataset contained 1728 images with 6297 annotated food instances across 16 classes. The final distribution remained non-uniform because the objective of balancing was to improve minority-class representation without artificially forcing equal class frequencies.

The class distribution demonstrates the natural variation in food-instance frequency within the released dataset. High-frequency classes such as rice and salad represent commonly occurring cafeteria meal components, whereas lower-frequency classes provide challenging examples for recognition and segmentation models.

### 3.3 Distribution across dataset splits

To evaluate whether the dataset partitioning process maintains consistent class representation, the distribution of annotated food instances across the training, validation, and test subsets was analyzed. Fig. 4 presents the percentage distribution of each food class across the three subsets. The similar distribution patterns among training, validation, and test partitions indicate that the dataset split maintains comparable class representation without substantial distribution shifts. This supports reliable model training and unbiased evaluation.

**Fig. 4:**
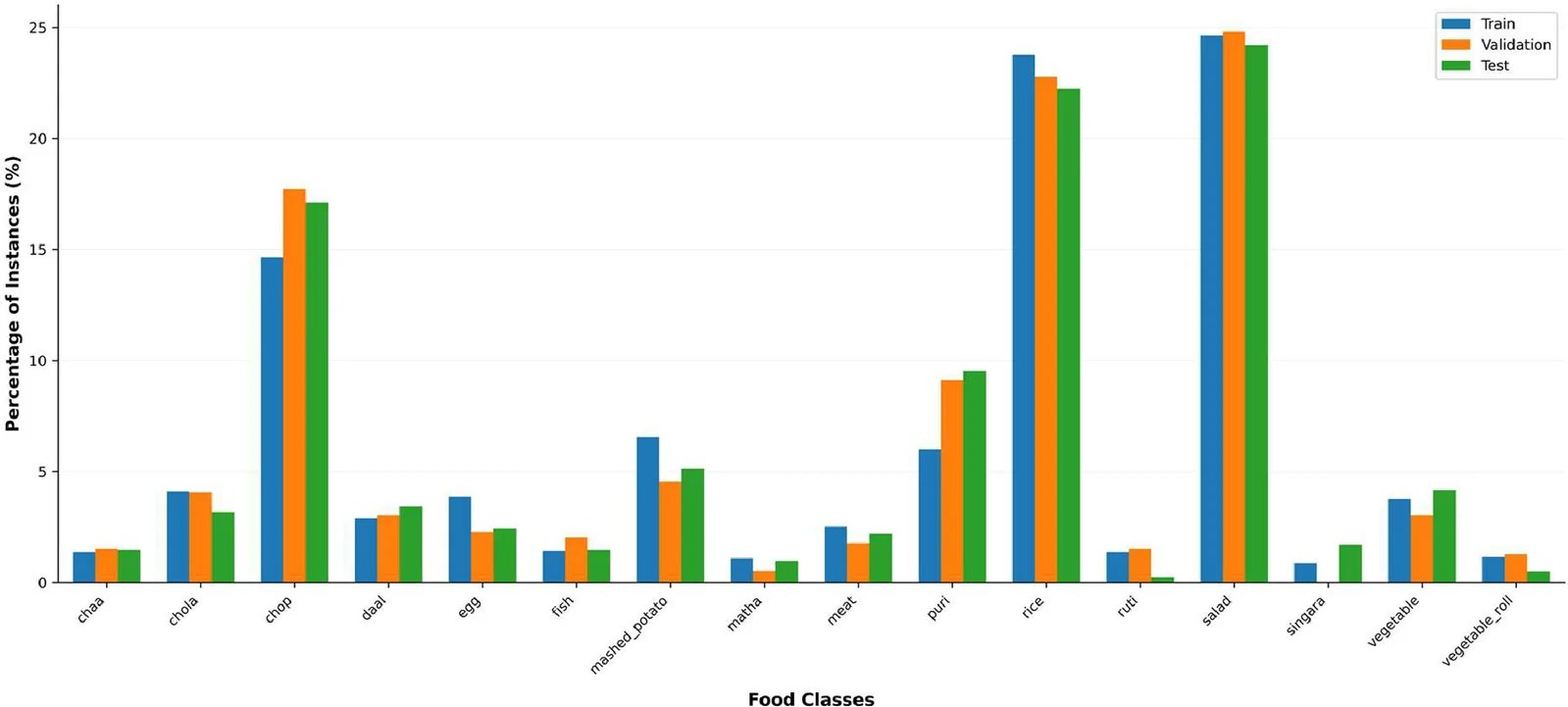
Split-wise distribution of annotated food instances across the PUST Cafeteria Dataset.

### 3.4 Annotation files and data formats

The dataset package includes processed meal-platter images and corresponding annotations for object detection and instance segmentation. All processed images are provided in JPG format at a standardized resolution of 640 × 640 pixels. The package contains three parallel data branches: YOLO object detection, YOLO instance segmentation, and COCO-format annotations. Each branch is organized into training, validation, and test subsets. In the YOLO object-detection branch, each annotated food instance is represented by a numerical class identifier and normalized bounding-box coordinates. In the YOLO instance-segmentation branch, each instance is represented by a class identifier followed by normalized polygon vertex coordinates delineating the visible food region. Images containing multiple food items include a separate annotation record for each distinguishable instance. The COCO branch contains the corresponding images and a partition-specific _annotations.coco.json file. Each JSON file organizes image, category, and instance-annotation records in a structure compatible with COCO-based computer-vision workflows. The three branches are organized using the same 16-class food taxonomy and training, validation, and test partition structure, supporting reuse across different object-detection and instance-segmentation frameworks. Table 4 summarizes the data components and annotation formats. Representative examples of the bounding-box and polygon-based annotations are presented in Fig. 5.

**Table 4:** Data components and annotation formats included in the dataset.

Data component	File format	Content
Processed meal-platter images	JPG	Meal-platter images standardized to 640 × 640 pixels
YOLO object-detection annotations	TXT	Class identifiers and normalized bounding-box coordinates
YOLO instance-segmentation annotations	TXT	Class identifiers and normalized polygon vertex coordinates
COCO-format annotations	JSON	Image, category, and instance-annotation records organized by dataset partition

**Fig. 5:**
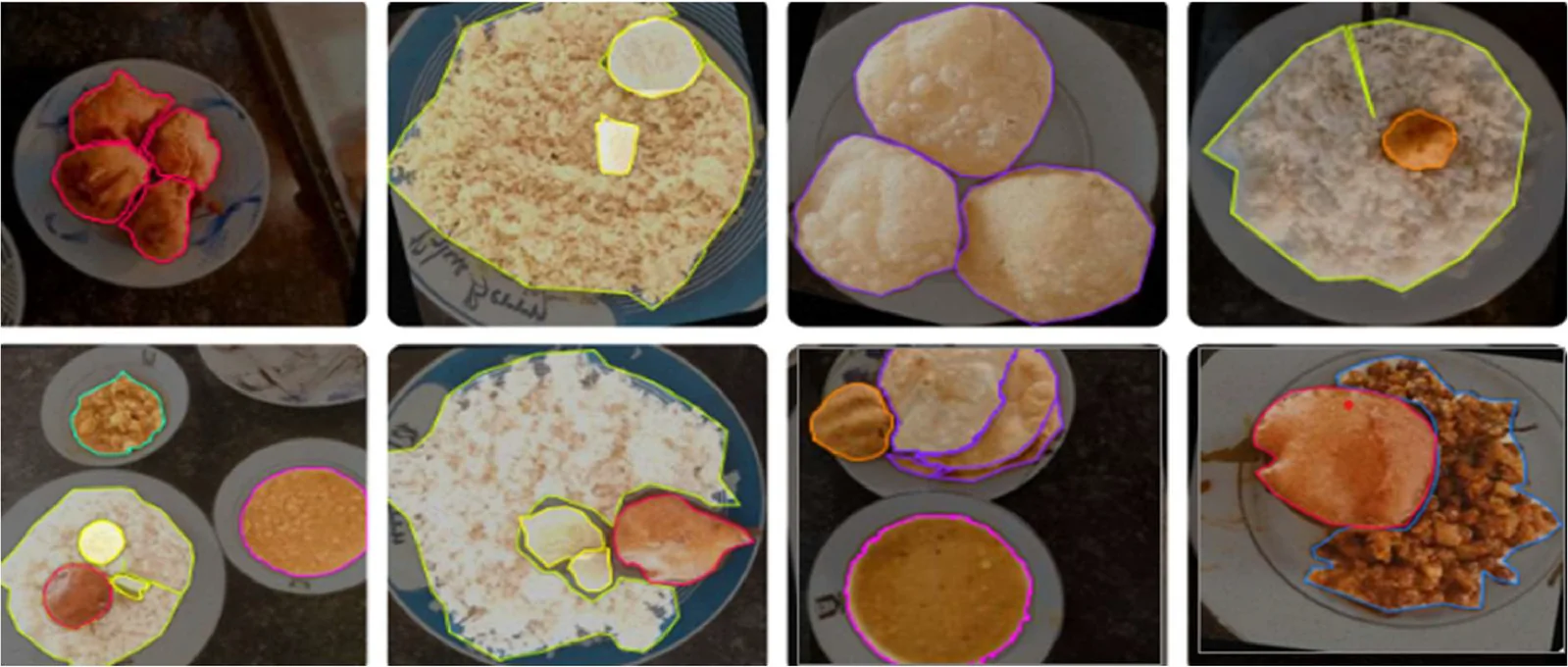
Examples of bounding-box and polygon-based instance annotations.

### 3.5 Dataset organization and directory structure

The dataset is organized into three primary components: 01_raw_original_images, 02_processed_dataset, and 03_metadata, together with the accompanying README.md and LICENSE.txt files. This structured organization facilitates transparent dataset reuse, annotation accessibility, and reproducible experimentation. The 01_raw_original_images directory contains the 720 original meal-platter photographs collected under natural cafeteria conditions. These images are provided in JPG format and represent the unprocessed source collection used for annotation and subsequent dataset preparation. The 02_processed_dataset directory contains the final processed dataset comprising 1728 images with 6297 annotated food instances across 16 classes. The processed dataset is organized into three parallel annotation branches: coco, yolo_detect, and yolo_seg. These branches represent alternative annotation formats of the same processed image collection and should not be interpreted as separate datasets. Each annotation branch follows the same partition structure consisting of train (1512 images), valid (108 images), and test (108 images) subsets, where valid denotes the validation subset. The coco branch contains partition-specific image directories and corresponding _annotations.coco.json files, providing COCO-compatible image, category, and instance-level annotations for object detection and instance segmentation workflows. The yolo_detect branch provides YOLO-compatible bounding-box annotations, including separate images and labels directories for each partition and a data.yaml configuration file defining dataset paths and the 16-class taxonomy. The yolo_seg branch contains corresponding polygon-based instance segmentation annotations in YOLO segmentation format, with parallel images and labels directories. The 03_metadata directory contains supporting documentation and machine-readable metadata files, including class_names.csv, dataset_summary.csv, image_source_mapping_final.csv, instances_before_augmentation.csv, instances_after_augmentation.csv, and device_information.txt. These files provide information regarding class taxonomy, dataset statistics, image-source lineage, augmentation-related instance distribution, and image-acquisition details to improve dataset interpretation and reproducibility. The accompanying README.md file provides detailed information about dataset contents, annotation formats, directory organization, preprocessing workflow, and usage instructions. The LICENSE.txt file specifies the conditions for dataset reuse and distribution. The complete directory hierarchy and organization of the released dataset are illustrated in Fig. 6.

**Fig. 6:**
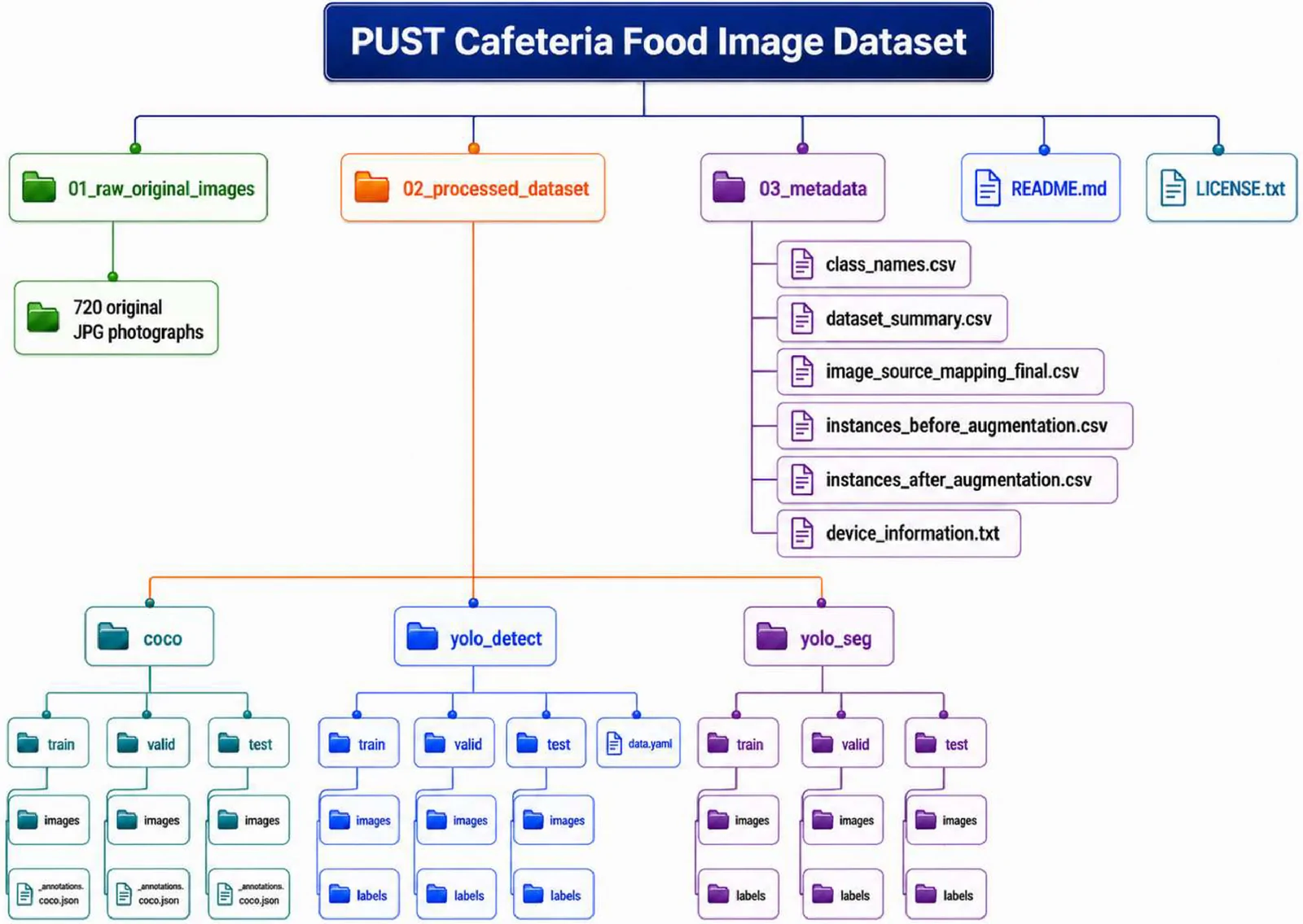
Directory structure of the dataset.

## 4 Experimental Design, Materials and Methods

The PUST Cafeteria Food Image Dataset [8] was developed through six sequential stages: image collection, food-instance annotation, original-image-level dataset partitioning, image preprocessing and training-only augmentation, light class balancing and dataset refinement, and preparation of the released annotation formats. The 720 original meal-platter photographs were partitioned into training, validation, and test subsets before augmentation was applied. Augmentation and subsequent light class balancing were performed only within the training subset, while the validation and test subsets remained unchanged. The complete dataset-development workflow is summarized in Fig. 7, and the corresponding procedures are described in 4.1, 4.2, 4.3, 4.4, 4.5.

**Fig. 7:**
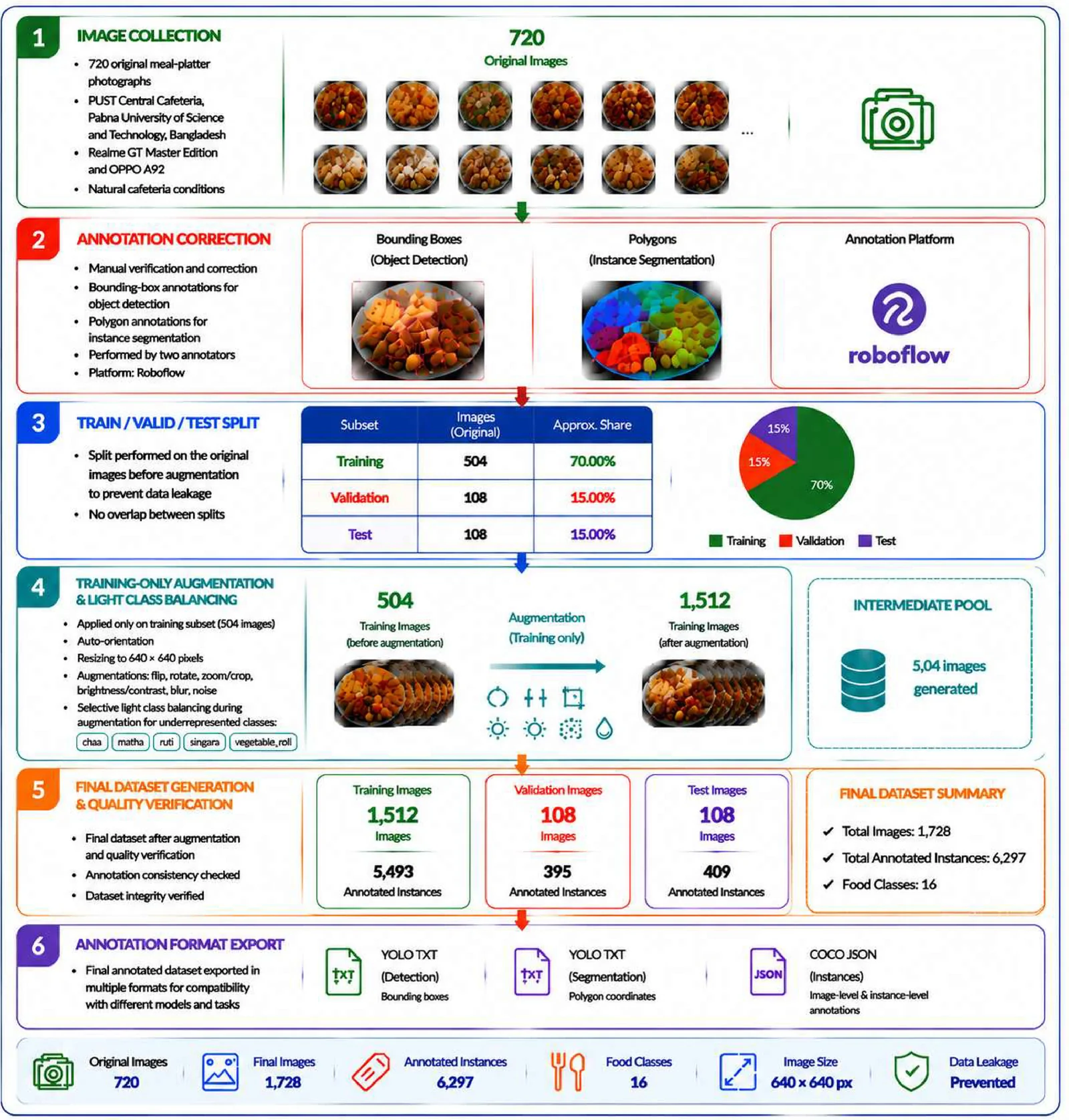
Workflow used to prepare the PUST Cafeteria Food Image Dataset.

### 4.1 Image collection and acquisition conditions

The 720 original meal-platter photographs were collected between 1 August and 5 September 2025 at the Central Cafeteria of Pabna University of Science and Technology, Pabna-6600, Bangladesh. Images were captured during routine cafeteria operations using the rear-facing cameras of two smartphones: Realme GT Master Edition and OPPO A92. Permission for data collection was obtained from the cafeteria authority. No controlled imaging setup or standardized capture protocol was applied during image acquisition. Variations in camera position, viewing angle, camera-to-platter distance, illumination conditions, background, plate configuration, and food arrangement were intentionally retained to represent realistic cafeteria environments rather than laboratory-controlled conditions. The collected images included both single-item and multi-item meal platters, containing adjacent, overlapping, and partially occluded food instances. These natural variations introduce realistic challenges for food detection and segmentation tasks while improving the practical relevance of the dataset. Details of annotation, preprocessing, augmentation, dataset partitioning, and quality-control procedures are described in 4.2, 4.3, 4.4, 4.5.

### 4.2 Food-instance annotation procedure

The 720 original meal-platter photographs were manually annotated using the Roboflow platform by two annotators. Each identifiable food item was assigned one of the 16 numerical class identifiers listed in Table 2. For multi-item meal platters, each visually distinguishable food component was annotated as an individual food instance. Two complementary annotation formats were generated: bounding-box annotations for object detection and polygon annotations for instance segmentation. Bounding boxes were drawn around the visible extent of each food instance, while polygon vertices were manually placed along the visible boundaries of the corresponding food regions. In images containing adjacent, overlapping, or partially occluded food items, separate annotations were created whenever individual instances could be visually distinguished. Polygon masks represented only the visible portion of each food item without estimating hidden regions. The annotation process involved independent manual labeling followed by cross-checking between the two annotators to ensure consistency in class assignment, bounding-box placement, polygon delineation, and image-label correspondence. Ambiguous or unclear food regions were reviewed carefully before final inclusion. The finalized annotations were verified prior to generating the released YOLO-compatible TXT and COCO JSON files. Representative examples of bounding-box and polygon-based annotations are presented in Fig. 5. Further dataset verification, preprocessing, augmentation, and partitioning procedures are described in 4.3, 4.4, 4.5.

### 4.3 Image preprocessing, training-only data augmentation, and light class balancing

The images were auto-oriented using the available image metadata and resized to 640 × 640 pixels to standardize the input dimensions. Data augmentation was applied exclusively to the training subset to increase geometric and photometric variability while preventing data leakage. The applied augmentation operations included horizontal and vertical flipping, random zooming/cropping, rotation, brightness and saturation adjustment, Gaussian blur, and salt-and-pepper noise. The augmentation parameters are summarized in Table 5.

**Table 5:** Image preprocessing and augmentation settings.

Processing operation	Applied setting
Auto-orientation	Orientation corrected using available image metadata
Resizing	640 × 640 pixels
Flipping	Horizontal and vertical
Random zoom/cropping	0–8%
Rotation	−15° to +15°
Brightness adjustment	−14% to +14%
Saturation adjustment	−25% to +25%
Gaussian blur	Up to 1 pixel
Salt-and-pepper noise	Up to 1.09% of pixels

The original dataset was divided at the image level into training, validation, and test subsets containing 504, 108, and 108 images, respectively. Augmentation was performed exclusively on the training subset, expanding it from 504 to 1512 images. Augmentation operations were applied across all 16 food classes to increase training diversity. In addition, selective augmentation was performed for five relatively underrepresented classes as a light class-balancing strategy: chaa (Tea), matha (Buttermilk), ruti (Flatbread), singara (Singara), and vegetable_roll (Vegetable roll). The changes in training-instance representation for these underrepresented classes before and after the combined augmentation and balancing process are presented in Table 6.

**Table 6:** Changes in training-instance distribution after augmentation and light class balancing for underrepresented classes.

Class ID	Dataset label	Before augmentation and balancing (Train instances)	After augmentation and balancing (Train instances)	Increase in instances
0	Chaa	24	75	51
7	Matha	18	60	42
11	Ruti	25	75	50
13	Singara	16	48	32
15	vegetable_roll	21	63	42
Total		104	321	217

Following augmentation, selective class balancing, annotation verification, and quality refinement, the final processed dataset contained 1728 images with 6297 annotated food instances across 16 classes, consisting of 1512 training images, 108 validation images, and 108 test images. The validation and test subsets were kept unchanged throughout the process to ensure unbiased evaluation and prevent data leakage.

### 4.4 Final dataset organization and annotation-format preparation

The final processed dataset containing 1728 images with 6297 annotated food instances across 16 classes was prepared in three parallel annotation formats: COCO, YOLO_detect, and YOLO_seg. These formats represent different annotation representations of the same dataset and follow the same train, valid, and test structure. The YOLO_detect branch contains bounding-box annotations in TXT format with a data.yaml configuration file, while the YOLO_seg branch provides polygon-based instance segmentation annotations in TXT format. The COCO branch includes JSON annotation files containing image, category, and instance-level information for object detection and segmentation tasks. The overall organization of the released dataset structure is illustrated in Fig. 6.

### 4.5 Quality control and dataset verification

Manual quality control was performed throughout the annotation and dataset refinement processes. Each image-annotation pair was reviewed to ensure correct food-class assignment, accurate bounding-box placement, proper polygon delineation of visible food regions, and consistency between image files and annotation records. Images containing adjacent, overlapping, or partially occluded food items were further examined to confirm that visually distinguishable items were annotated as separate instances. After preparing the final annotation formats, the dataset structure was verified to ensure the correct organization of the COCO, YOLO_detect, and YOLO_seg branches. Each branch was checked for consistent train, valid, and test partitions, with corresponding image and annotation files properly aligned. The YOLO branches were verified for image-label consistency and the presence of the required configuration files, while COCO annotations were checked for valid image, category, and instance records. The final dataset records were cross-validated to confirm consistency between images, annotations, and class taxonomy. The released dataset contains 1728 images with 6297 annotated food instances across 16 classes, maintaining identical class identifiers across all annotation formats.

## Limitations

Although the PUST Cafeteria Food Image Dataset [8] provides a realistic benchmark for food recognition, object detection, and instance segmentation, several limitations should be considered. The dataset was collected from a single university cafeteria within a limited acquisition period using two smartphone cameras. Therefore, the visual characteristics of the dataset reflect the specific food items, serving practices, plate arrangements, and environmental conditions of that cafeteria. Models trained on this dataset may require further validation and adaptation before deployment in other food-service environments with different menus, cultures, or imaging conditions. The dataset includes 16 food classes with a markedly non-uniform class distribution. Although selective training augmentation and light class balancing were applied to improve the representation of less frequent classes, substantial frequency disparities remain. In the final released dataset, majority classes such as salad (1550 instances) and rice (1486 instances) are considerably more represented than several minority classes. This imbalance may affect model evaluation because aggregate performance measures can be dominated by majority classes, whereas class-specific precision, recall, F1-score, and related metrics for minority classes may be less stable because of their limited representation. Therefore, model performance should be evaluated using both overall and class-wise metrics, and results for minority classes should be interpreted with appropriate caution. Additionally, augmented samples increase image variability but cannot fully replace the diversity obtained from independently collected meal scenes. For instance segmentation, polygon annotations represent only the visible regions of food items and do not estimate hidden areas affected by overlap or occlusion. Although annotation quality was manually reviewed, an independent multi-annotator agreement analysis was not performed. Furthermore, the dataset provides visual annotations only and does not include additional information such as food weight, portion size, ingredients, preparation methods, pricing, or nutritional composition. Therefore, applications involving automated billing, portion estimation, calorie prediction, or dietary assessment require integration with external validated information sources. Future work may focus on expanding the dataset across multiple locations, increasing class diversity, and incorporating additional food-related metadata.

## CRediT Author Statement

**Marufa Yeasmin:** Conceptualization, Methodology, Investigation, Data curation, Formal analysis, Visualization, Writing – original draft. **Md. Razu Ahmed:** Conceptualization, Methodology, Investigation, Data curation, Validation, Visualization, Writing – review & editing. **Protiva Rani Kundu:** Conceptualization, Investigation, Data curation, Visualization, Writing – review & editing. **Jannatul Mauya:** Investigation, Validation, Writing – review & editing. **Md. Shamim Reza:** Conceptualization, Methodology, Validation, Supervision, Project administration, Writing – review & editing.

## Ethics Statement

The dataset comprises photographs of prepared meal platters. The work did not involve human participants or animals and did not include the collection of personal, clinical, or other sensitive information. Permission to photograph the meal platters at the Central Cafeteria of Pabna University of Science and Technology was obtained from the responsible cafeteria authority. No personally identifiable information is included in the dataset; therefore, informed consent was not applicable.

## Declaration of Generative AI and AI-assisted Technologies in the Manuscript Preparation Process

During the preparation of this work, the authors used ChatGPT (OpenAI) for language refinement, manuscript organization, and assistance in preparing explanatory workflow and dataset-structure diagrams. After using this tool, the authors reviewed and edited the content as needed and take full responsibility for the content of the publication.

## Funding

This work was partially supported by a research grant fromPabna University of Science and Technology (PUST) and by the National Science and Technology (NST) Fellowship of the Ministry of Science and Technology, Government of the People’s Republic of Bangladesh.

## Data Availability

The data described in this article are openly available in Mendeley Data under the title PUST Cafeteria Food Image Dataset: Real-World Bangladeshi Meal-Platter Images with Bounding-Box and Polygon Annotations, Version 2, doi:10.17632/fn6yhzjz83.2 [8].
